# The fetal programming of telomere biology hypothesis: an update

**DOI:** 10.1098/rstb.2017.0151

**Published:** 2018-01-15

**Authors:** Sonja Entringer, Karin de Punder, Claudia Buss, Pathik D. Wadhwa

**Affiliations:** 1 Charité—Universitätsmedizin Berlin, corporate member of Freie Universität Berlin, Humboldt-Universität zu Berlin, and Berlin Institute of Health (BIH), Institute of Medical Psychology, Berlin, Germany; 2 Department of Psychiatry and Human Behavior, University of California, School of Medicine, Irvine, CA, USA; 3 Department of Obstetrics and Gynecology, University of California, School of Medicine, Irvine, CA, USA; 4 Department of Pediatrics, University of California, School of Medicine, Irvine, CA, USA; 5 Department of Epidemiology, University of California, School of Medicine, Irvine, CA, USA; 6 Development, Health and Disease Research Program, University of California, School of Medicine, Irvine, CA, USA

**Keywords:** telomere, telomerase, development, fetal programming, health and disease risk

## Abstract

Research on mechanisms underlying fetal programming of health and disease risk has focused primarily on processes that are specific to cell types, organs or phenotypes of interest. However, the observation that developmental conditions concomitantly influence a diverse set of phenotypes, the majority of which are implicated in age-related disorders, raises the possibility that such developmental conditions may additionally exert effects via a common underlying mechanism that involves cellular/molecular ageing–related processes. In this context, we submit that telomere biology represents a process of particular interest in humans because, firstly, this system represents among the most salient antecedent cellular phenotypes for common age-related disorders; secondly, its initial (newborn) setting appears to be particularly important for its long-term effects; and thirdly, its initial setting appears to be plastic and under developmental regulation. We propose that the effects of suboptimal intrauterine conditions on the initial setting of telomere length and telomerase expression/activity capacity may be mediated by the programming actions of stress-related maternal–placental–fetal oxidative, immune, endocrine and metabolic pathways in a manner that may ultimately accelerate cellular dysfunction, ageing and disease susceptibility over the lifespan. This perspectives paper provides an overview of each of the elements underlying this hypothesis, with an emphasis on recent developments, findings and future directions.

This article is part of the theme issue ‘Understanding diversity in telomere dynamics’.

## 1. Overview

**Figure 1.**
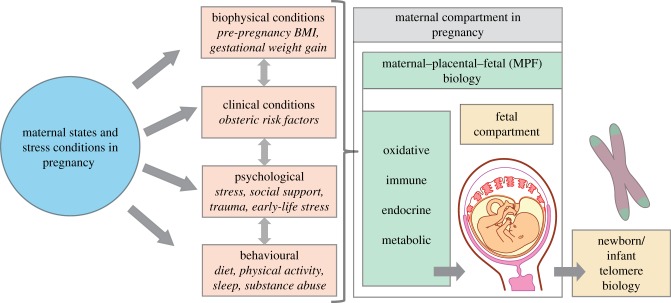
The fetal programming of telomere biology hypothesis: a conceptual framework.

This perspectives paper articulates a transdisciplinary framework that underlies the fetal programming of telomere biology hypothesis primarily in the context of humans. Our model, depicted in figure 1, proposes that (*a*) intrauterine life represents a particularly sensitive time period when the effects of maternal states and conditions around conception and across pregnancy may be transmitted to the developing embryo/fetus; (*b*) transmission occurs primarily via the effects of various maternal biophysical, clinical, psychological and behavioural states and conditions on stress-related maternal–placental–fetal (MPF) oxidative, immune/inflammatory, endocrine and metabolic pathways that participate in the process of fetal programming of health and disease risk; and (*c*) the initial setting and function of the offspring telomere biology system—telomere length and telomerase expression and activity capacity—exhibits developmental plasticity and represents a key cellular target of such programming, with important implications for long-term health and susceptibility for common age-related disorders.

We begin this paper with brief overviews of the concept of fetal programming of health and disease risk and the role of telomere dynamics in humans. We then proceed to discuss the importance of the initial setting of the telomere biology system. Next, we discuss the concept of fetal programming specifically in reference to the telomere biology system. In the context of inter-generational transmission, we identify stress-related MPF gestational biology as a leading candidate pathway of interest, and we describe how variation in stress-related MPF gestational biology may impact fetal developmental trajectories. While this perspectives paper is focused on humans, we note that there are related studies on a wide range of taxa, including non-primate mammals, birds, reptiles and fish, that may not have the same telomere dynamics across the lifespan as humans, but nevertheless make important contributions towards understanding the role of the telomere system in linking developmental conditions in early life with life-history trajectories, health and longevity [1–3]. We conclude by articulating current knowledge gaps and future research directions. We have described various aspects of this formulation in previous papers [4–7], and the present perspective updates our framework, with an emphasis on a discussion of recent findings in the literature and implications for future research directions.

## 2. The concept of fetal/developmental programming

The origins of many, if not all, complex common age-related disorders that confer the major societal burden of disease can be traced back to developmental processes in embryonic, fetal and early postnatal life. At the individual level, the likelihood of developing a complex common disorder is a joint function of cumulative risk exposures (e.g. excess caloric intake, infection, stressful life events) and susceptibility to these exposures (e.g. as reflected in the wide inter-individual variation in biological responses to the particular risk exposure(s) in question) [5,8,9]. Development is a plastic, context-dependent process, wherein a range of different phenotypes can be expressed from a given genotype. Thus, contrary to the conventional paradigm that asserts individual susceptibility is determined primarily by DNA sequence variation, it is apparent that susceptibility for complex common disorders is more profoundly determined by the dynamic interplay between genetic make-up and environmental conditions, particularly during the earlier periods of life [10,11]. The embryo/fetus seeks, receives and responds to, or is acted upon by, its environment during sensitive periods, resulting in structural and functional changes in cells, tissues and organ systems. These changes may, either independently or through interactions with subsequent developmental processes and environments, have major consequences for health and disease susceptibility over the individual's lifespan [10,12,13]. These concepts have variously been referred to as the fetal or developmental origins of health and disease risk. The process is adaptive from an evolutionary perspective, but may in some instances confer increased susceptibility at the individual level, particularly when there is a discrepancy between the nature of environmental conditions during development and those during later stages of life. Also, except in extreme cases, fetal programming does not, *per se*, ‘cause’ disease, but, instead, may influence an individual's susceptibility or propensity for disease(s) in later life, for instance, by shaping responsivity to subsequent endogenous and exogenous conditions.

The majority of studies on mechanisms underlying fetal/developmental programming effects have focused primarily on processes that are specific to cells, organs or phenotypes of interest (e.g. mechanisms within the adipocyte, haematopoietic cell, brain, pancreas, liver, etc.). However, the observation that exposure to adverse intrauterine conditions concomitantly influences a diverse set of phenotypes, coupled with the fact that the majority of these phenotypes are implicated in common age-related disorders, raises the possibility that prenatal and early postnatal conditions may also additionally (not instead) exert effects via a common underlying mechanism, and that such a mechanism may involve cellular ageing–related molecular processes. In this context, we submit that the telomere biology system represents a candidate mechanism of particular interest [4].

## 3. The importance of the telomere biology system: a brief overview

Telomere biology is a highly evolutionarily conserved system that plays a central role in maintaining the integrity of the genome and cell [14]. Telomere biology refers to the structure and function of two entities—*telomeres*, non-coding double-stranded repeats of guanine-rich tandem DNA sequences and shelterin protein structures that cap the ends of linear chromosomes, and *telomerase*, the reverse transcriptase enzyme that adds telomeric DNA to telomeres.

### (a) Telomeres

Telomeres protect chromosomes from mistaken recognition by the DNA damage-repair system as DNA breaks. Because DNA polymerase is unable to fully replicate the 3′ end of the DNA strand, telomeres lose approximately 30–150 base pairs (bp) with each cell division [15]. Eventually, telomeres reach a critical short length, resulting in decreased recruitment of shelterin proteins to form the protective internal nucleotide loops, which, in turn, leads to cellular senescence or apoptosis. Once cells become senescent, they exhibit a variety of genetic and morphological changes, such as chromosomal fusion, activation of DNA damage checkpoint responses and genome instability, that result in loss of tissue function. Senescent cells also produce inflammatory mediators that affect neighbouring cells, leading to further damage within tissues and organs that accumulates over time. Thus, as individuals age, they acquire more senescent cells, accompanied by various age-related pathologies [14]. Moreover, recent important discoveries suggest that the integrity of telomeres affects not only the replicative capacity of the cell, but also underlies other changes that enforce a self-perpetuating pathway of global epigenetic changes to affect the integrity of overall chromatin structure (DNA folding) that protects against senescence and cellular ageing [16–18]. Thus, this is how a reduction in telomere length and a steeper telomere attrition rate relate not only to longevity, but also to earlier onset and more rapid progression of common age-related disorders.

### (b) Telomerase

Telomerase is a ribonucleoprotein enzyme consisting of an RNA component (TR or TERC) and a catalytic protein domain (TERT). Conventional DNA polymerase machinery is unable to fully replicate the ends of linear chromosomes. Telomerase uses its own template to add short TG-rich repeats to chromosome ends, thus attenuating their gradual erosion at each round of replication [19]. Typically, telomerase activity is diminished or absent in most adult somatic cells, with the exception of cells with a strong potential for division, such as stem cells and active lymphocytes [20]. The selective reduction of telomerase expression makes senescence inevitable by placing an upper bound on cellular lifespan [21]. Moreover, telomerase not only regulates telomere length but also preserves healthy cell function. Loss of telomerase affects chromatin configuration and impairs the DNA damage response. Telomerase also promotes proliferation of resting stem cells and directly modulates crucial developmental signalling pathways [22]. Through telomere capping and maintenance, telomerase plays a particularly important role in cellular proliferation capacity and survival under conditions of cellular stress. Finally, telomerase also performs an extra-nuclear role to co-localize with mitochondria to protect mitochondrial DNA, decrease oxidative stress, and improve energy production and cellular function [23–25]. Thus, a diminished capacity to express telomerase leads to more rapid telomere attrition over time, impaired DNA damage responses and impaired cellular energetic function.

If telomere shortening represents the clock ticking forwards on cells' limited lifespan, telomerase can reverse or slow this clock, making the two an intricately inter-dependent, dynamic system.

### (c) Telomerase maximal expression/activity capacity

The majority of human studies of telomere biology have focused largely on the telomere length component of the system; relatively few studies have considered the role of telomerase. The importance of including measures of telomerase derives from the understanding that its expression and activity constitutes a *critical* and *complementary* (i.e. non-redundant) component of the functional integrity of the telomere biology system [26–28]. For example, in a yeast single-cell model system, Blackburn and colleagues showed that well before critical telomere shortening occurs, telomerase is continuously required to respond to transient DNA replication stress, and that a lack of telomerase accelerates otherwise normal ageing [29]. The few studies that have included measures of telomerase have typically measured telomerase expression or activity under basal conditions or in terms of its acute (short-term) response to systemic challenges (see, e.g. [30]). However, given the limitations in the quantification and interpretation of basal telomerase data (because telomerase is typically not expressed, or expressed only at very low levels, in most resting cells [20]; telomerase levels may vary as a function of cell-cycle stage and other factors [31]; and differences or changes in telomerase may reflect either the direct effects of states or conditions that stimulate telomerase expression, or the secondary (compensatory/counter-regulatory) adaptations to states or conditions that reduce telomere length [32]), we have recently proposed the use of an *in vitro* measure of *maximal telomerase activity capacity* of human leucocytes in response to mitogen challenge (*mTAC*). We have determined that this measure empirically meets the key criteria to represent a potentially useful individual difference construct in this context (i.e. adequate within-subject stability and across-subject variability; see de Punder K *et al.* [33]).

### (d) Role of telomere biology in ageing, disease susceptibility and longevity

A substantial and largely convergent body of human and animal research has linked shortened telomeres and/or reduced telomerase expression to several age-related risk factors, diseases [34–43] and longevity [44–48]. For example, relatively recent papers have reported associations in humans between telomere length and increased mortality risk [49,50], cardiovascular disease [51,52], diabetes and the metabolic syndrome [53,54], suboptimal brain anatomy [55], impaired cognitive function [56], psychiatric disorders [57] including depression [58–61] and PTSD [62], toxicity of chemotherapy [63] and the robustness of the immune system [64,65]. Shorter telomere length also has been associated in embryonic stem cells with unstable differentiation [66], and in haematopoietic progenitor cells with reduced proliferative reserve [67]. Lastly, a *causal* role for the telomere biology system in human health and disease risk is suggested by findings from several organ transplantation studies that report recipient survival time and disease progression-related outcomes are a function of donor and not recipient telomere length [68–71]. Despite the evidence linking short telomeres with increased disease risk in humans, the above-mentioned association studies do not prove causality, and the involvement of short telomeres in ageing-related processes has been questioned in some non-human model systems [72,73]. Further experimental and mechanistic studies are clearly warranted.

Nevertheless, to summarize, it appears that telomere homeostasis in various cell types, including the germ line, stem cells and proliferating as well as post-mitotic tissue, may serve as a fundamental integrator and regulator of processes underlying cell genomic integrity, function, ageing and senescence, which, in turn, may have major implications for health and disease susceptibility for complex common disorders.

### (e) Determinants of variation in the telomere biology system

In humans, telomere length and telomerase expression and activity is associated with a host of socio-demographic, biophysical, clinical, biological, behavioural and psychosocial states and conditions, including age, sex, socio-economic status, race/ethnicity, body mass index, infection, diet/nutrition, physical activity, sleep, stress and social relationships. A detailed description is outside the purview of the current paper, but see [74–85] for recent studies and reviews.

#### (i) Role of stress and stress biology

Most of the above-mentioned states and conditions associated with unfavourable alterations in telomere dynamics reflect forms of social disadvantage or adversity characterized by high levels of stress. Epel, Blackburn and co-workers were the first to propose and demonstrate a link between chronic psychosocial stress burden and telomere biology [86]. This relationship has since been replicated in several [77,87–89] but not all studies [90,91]. Exposure to severe psychological trauma or other psychopathological conditions also has been linked to telomere biology [92–96]. A recently published meta-analysis on one specific component of psychosocial stress—perceived stress—suggests there is a significant association across studies, but of relatively modest magnitude [77]. Furthermore, some studies have suggested that lifestyle interventions that attenuate stress may increase telomerase activity and slow down cellular ageing [82,97,98].

A substantial body of animal and human research has elucidated the role of stress-related biological processes in mediating the effects of stress and unhealthy behaviours on the regulation of telomere dynamics, including oxidative stress, inflammation, stress hormones and metabolic processes, as discussed below.

*Oxidative stress:* Telomeres have a high content of guanine residues, and these are particularly sensitive to oxidative damage. Thus, oxidative stress potently accelerates telomere shortening, decreases telomerase activity and induces senescence or apoptosis via DNA damage-induced activation of the *p53* pathway [96,99–104]. Oxidative stress also can induce the nuclear export of TERT to the cytosol and into the mitochondria, thereby decreasing nuclear and total telomerase activity [100]. The effects and mechanisms by which oxidative stress at the cellular and organismal level impacts telomeres are discussed in greater detail by Monaghan & Ozanne [28].

*Inflammatory mediators:* Biological mediators of inflammation such as C-Reactive Protein, Interleukin (*IL*)-6 and tumour necrosis factor (TNF)-α have been linked to telomere shortening [105–107] and T-cell senescence [108]. Activated immune cells such as T-cells express high telomerase levels. The upregulation of telomerase prevents immune cell senescence and facilitates a rapid and profound clonal cell expansion. However, the level of telomerase expression is not sufficient to indefinitely prevent telomere shortening and senescence [109]. For instance, continuous antigen challenge has been shown to produce accelerated telomere shortening and premature senescence in human cytotoxic T-cells [109]. In human lymphocytes, TNF-α administration *in vitro* induced the nuclear translocation of TERT and increased telomerase activity via the nuclear factor kappa B (NF-κB) signalling pathway [110]. Conversely, TERT regulates the expression of a subset of NF-κB-dependent genes [111,112]. The observation that TERT binds to the NF-κB p65 subunit and is recruited to a subset of NF-κB target gene promoters such as those of IL-6 and TNF-α suggests that telomerase can provide a feed-forward loop for the immune system by stimulating NF-κB-dependent gene expression [112].

*Stress hormones:* In humans, several measures of the activity of the hypothalamic–pituitary–adrenal stress axis and its adrenal end product, cortisol, including higher production (overnight urinary free cortisol levels), dysregulation of feedback sensitivity (disruption of the diurnal cortisol rhythm) and greater reactivity (higher acute stress-induced cortisol responses) have been linked to shorter telomeres [113–115]. We recently reported that immune cells from individuals who exhibit greater cortisol responses to stress have a lower capacity to induce telomerase activity (mTAC) (see de Punder K *et al.* [33]). Consistent with this finding, exogenous cortisol exposure has been shown to inhibit telomerase production in mitogen-stimulated human T-cells [116]. Long-term stress exposure also is known to induce oxidative stress and inflammation [117], both of which accelerate telomere shortening (as discussed above).

*Metabolic processes*: Metabolic processes related particularly to lipid and glucose–insulin physiology have been shown to regulate telomere dynamics. Evidence across several studies has linked the intake of total fat or specific fatty acids with leucocyte telomere length (LTL; summarized in [118]). A recent and large cross-sectional study demonstrated an association between key metabolic biomarkers (high-density lipoprotein (HDL) cholesterol and triglycerides) and LTL after controlling for the effects of socio-demographic factors, health-related behaviours and immune cell-type composition [119]. Another recent study of metabolomic profiling in a large group of females reported that specific alterations in lipid metabolism that indicate changes in cell membrane composition were associated with LTL, and also with higher blood pressure, HDL cholesterol levels, and poorer lung, liver and kidney function [104]. In terms of glucose–insulin physiology, insulin resistance is associated with shorter LTL [34,37], and a more recent longitudinal study indicated that individuals with shorter LTL are more likely to develop insulin resistance later in life [120]. A study in rodents demonstrated a regulatory role for telomerase in glucose metabolism; telomerase-deficient mice exhibited impaired glucose metabolism and insulin secretion [39]. In addition, *in vitro* studies revealed an extra-nuclear function of TERT in glucose uptake in mouse skeletal muscle cells [121].

## 4. The importance of the initial setting of the telomere system

Telomere length, at any given age, is a joint function of the initial (newborn) setting of TL and the magnitude of TL attrition over time, which, in turn, is a function of the number of cell divisions (reflected by growth, age), exposure to oxidative and other forms of biological stress that reduce TL, and the counter-regulatory effect of telomerase expression and activity that attenuates TL reduction [4]. Studies in animals and humans converge to provide a strong rationale for the importance of newborn and infant telomere biology in long-term health and disease risk, as discussed below.

### (a) Animal studies

Animal models of telomere dynamics *over the lifespan* and *across generations* suggest that *initial TL* and the *TL attrition rate in early life* is (*a*) a better predictor of realized lifespan than TL and the TL attrition rate in later life [122–124], and (*b*) the effects of early-life TL persist over and beyond those of risk exposures in later life [122–124]. For example, a recent study on telomere dynamics in birds reported that early-life TL predicted lifespan, and its effect persisted and was substantially unchanged even after accounting for the effect of subsequent life course exposures such as chronic infection [124]. Another study of life-long (birth to death) patterns of LTL variation in sheep reported that LTL variation was significantly associated with longevity, and particularly that it was longer LTL *specifically during the first 2 years of life, but not later in life (during adulthood)*, that drove this observed association [125].

### (b) Human studies

We are not aware of any human studies that have prospectively tracked TL from birth until old age (with characterization of common age-related disorders) or death. However, findings from cohorts that have longitudinally tracked TL over time, and others that have estimated the heritability of age-related TL attrition, have collectively suggested that *it is the initial (early life) setting of the system that accounts for the largest proportion of its attributable effects on health and disease risk-related outcomes*. For example, a recent study of 4 longitudinal cohorts (*N* = 1156) with mean ages of 30, 31, 58 and 78 years at baseline and an average 12-year follow-up concluded that most of the inter-individual variation in adult LTL originated early in life, because the overwhelming majority of individuals maintained their LTL ranking and it remained unchanged over 6 decades of adult life [126]. A study of the age-related heritability of LTL dynamics using the same-sex twin model (355 MZ and 297 DZ twins aged 19–64 years at baseline with an average follow-up of 12 years) reported that the *early-life* environment was the main determinant of LTL variation throughout the life course (72%), an approximately 2.5-fold greater effect than that of heritability (28%) [127]. Another recent human study in newborns demonstrated that compared to newborns with normal TL, newborns with reduced TL at birth exhibited greater DNA damage at baseline and also upon exposure to a genotoxic challenge [128]. Lastly, although TL is known to differ across tissue types, the rate of age-dependent TL shortening in humans appears to be similar across different somatic tissues (leucocyte, skeletal muscle, skin and fat), suggesting that the observed TL differences between tissues are established in early life [129].

### (c) Conceptual considerations

Several recent papers have considered some of the implications of the above-described findings. Broadly, it appears that selection may have favoured short telomeres as a mechanism to protect against cancer, and may have favoured long telomeres as a protective mechanism against DNA damage and replicative senescence [130,131].

Firstly, as discussed in their paper on telomere dynamics and ageing-related diseases in humans, Aviv and Shay have questioned the commonly held premise that adult telomere length may be an active determinant in adult-onset disease [131]. They submit that the view of adult telomere length as a ‘clock’ or maker of risk for age-related disorders, whose pace is modified by the cumulative burden of stress-related biological exposures such as oxidative stress and inflammation, may be overlooking the facts that telomere length is not uniformly calibrated at ‘zero time’ across newborns (on the contrary, the *magnitude* of TL variation in newborns is similar to that observed in adults), and that telomere length at birth is the principal determinant of telomere length throughout the life course. They also point out that longitudinal studies indicate that adults characterized by intermediate phenotypes underlying common age-related disorders (e.g. more atherosclerotic burden and insulin resistance) have shorter LTL but do not exhibit any evidence of a higher rate of age-dependent LTL shortening. They suggest that the overall influence of telomere length dynamics during adulthood may be smaller than that of TL at birth and its dynamics prior to adulthood, and they highlight the importance of expanding telomere research to newborns and children to better understand the role of telomere biology in age-related disorders and the causes of its variation.

Secondly, Monaghan & Ozanne [28] have highlighted the significance particularly of telomere length loss rate in early life. They argue that if short telomeres have a causal role in suboptimal health, the same loss rate may have different consequences, depending on the initial telomere length setting. They underscore the need to obtain repeated measures from the same individual, particularly during the early-life period, because telomere dynamics might be differentially related to individual state at different life stages, and because differential mortality with respect to telomere length may alter variation in telomere length in different age categories.

Thirdly, as reviewed in §3e above, a range of health-related behaviours such as diet/nutrition, physical activity and sleep have been associated in adults with telomere length and telomerase expression and activity, leading to the premise that one mechanism by which such behaviours influence health and disease risk is via their effects on telomere dynamics. However, Bateson & Nettle [132] have recently proposed a provocative alternative. They argue it is plausible that individuals with short telomeres may be more likely to adopt specific health-related behavioural patterns (i.e. the selective adoption hypothesis), and they suggest selective adoption could occur either because telomere length directly affects behaviour, or because behaviour and telomere length may both be affected by a third variable, such as exposure to early-life adversity. Thus, this formulation, again, but for a different set of reasons, highlights the potential importance of the initial setting of the telomere system in health and disease risk.

## 5. Fetal programming of the telomere biology system

It appears that the initial setting and regulation of telomere homeostasis, including chromosomal telomere length and both the telomeric and extra-telomeric activities of telomerase, may be plastic and receptive to the influence of conditions during intrauterine or early postnatal life. The assumption that the initial setting of telomere length is largely under genetic (DNA base pair sequence variation) control has been challenged for the following reasons: First, although the heritability of TL is high, known genetic variants (across all candidate gene and GWAS studies to date) collectively account for only a small proportion of variation in TL (e.g. [133,134]). Second, the mother–offspring correlation in TL appears to be larger than the father–offspring correlation, regardless of the sex of the offspring [135]. Third, a recent study that examined the association of the weighted genetic scores of TL-related variants with telomere length in over 400 maternal and newborn (cord) blood samples found that the maternal genetic score was significantly associated with maternal TL, but that there was no significant association of the newborn genetic score (or any of its individual variants) with newborn TL. This finding suggests that currently identified genetic loci do not appear to influence the initial (newborn) setting of telomere length, but that they may play a role in telomere length modification during the life course [136]. Collectively, these observations, in conjunction with the understanding that heritability may overestimate genetic effects (because it includes maternal intrauterine effects), emphasize a major role for maternal and intrauterine effects in the initial setting of TL. In fact, as underscored recently by Dugdale & Richardson [137], accurate and meaningful measures of the heritability of telomere length can be derived only after fully understanding and accounting for the nature and timing of environmental effects. Furthermore, experimental and observational studies in animals and humans (discussed below in §§5b and 5c) suggest adverse intrauterine conditions such as stress, poor diet/nutrition and obstetric complications are associated with shorter offspring TL or reduced telomerase activity at birth and/or in childhood and adult life [4,138], thereby providing biological plausibility for the fetal/developmental programming hypothesis.

### (a) Developmental ontogeny of the telomere biology system

Telomerase is especially active in germ cells, presumably to ensure the maintenance and transmission of full-length chromosomes to offspring [139–141]. After fertilization, telomerase remains abundant in the blastocyst and during early embryonic stages, and then decreases with increasing gestational age and cellular differentiation [142,143]. In children and adults, telomerase is largely inactive in most tissues except rapidly proliferating tissues such as certain types of stem cells and active lymphocytes [20]. However, when stimulated to divide, many stem or stem-like cells in adults exhibit telomerase activity. This activity is sufficient to slow, but not prevent, telomere shortening [20].

Consistent with their high levels of telomerase activity, germ cells have significantly longer telomeres than somatic cells, possibly because of telomere elongation during maturation [144,145]. It appears from studies in animals that telomeres may first be elongated during early embryonic development [141,146]. Later, during the fetal period, human studies of tissue samples from abortuses and newborns suggest that telomere length remains stable (does not decline) across gestation, and also that it is comparable across most fetal tissues [147]. In newborns, TL is highly synchronized between white blood cells, umbilical artery and foreskin tissues, but there is high variability between individuals [148]. Among cord blood haematopoietic cells, correlations in TL between the different cell types also are very high [149]. After birth, infants show a rapid decrease in TL [150,151], corresponding with rapid growth and high turnover of immune cells in the process of developing acquired immunity [152]. We are aware of only one prospective study that quantified telomere shortening during the first few years of life. In this study of a relatively small number of subjects, LTL was assessed serially from birth until 3 years of age [150]. Collectively, the findings from cross-sectional and longitudinal studies suggest that telomere shortening is accelerated during the first years of life (approx. 270 base pairs per year), compared to early adulthood (approx. 60 bp year^−1^ at 20 years of age) and old age (approx. 26 bp year^−1^) [152]. Furthermore, there is considerable variation in the rate of telomere shortening among young children [150].

### (b) Determinants of the initial setting of the telomere biology system

Human and animal studies support the concept that the initial setting of the telomere system exhibits developmental plasticity and is influenced by various physiological, social, environmental and clinical conditions in early life. We have advanced the hypothesis that context- and time-inappropriate exposures to *physiological* stress mediators during the conceptional, embryonic, fetal and early postnatal periods of development may alter or programme the telomere biology system in a manner that accelerates cellular dysfunction, ageing and disease susceptibility over the lifespan [4,5]. We have proposed that the same stress-related biological processes that mediate the effects of a range of unfavourable conditions on telomere biology during adult life (reviewed above in §3e(i)) may also impact fetal programming of the telomere system during the development. These stress-related MPF oxidative, immune endocrine and metabolic processes represent a plausible mechanism in this context because (*a*) they are sensitive to an array of adverse physiological, social, environmental and clinical exposures (summarized in [9]; (*b*) they constitute some of the key signalling molecules between the fetal and maternal compartments during intrauterine development [153]; and (*c*) they may exert stable, long-term effects via epigenetic and other processes on the developing telomere biology system [4,11]. Moreover, it is possible that the effects of these stress-related biological processes on telomere biology during development may be stronger than those during adulthood, because the system is undergoing particularly rapid changes during this period (e.g. faster TL attrition rate) [131].

We note that compared to many other phenotypes and outcomes that have been examined in the context of the process of fetal programming of health and disease risk, there are a relatively small number of studies to date that have addressed telomere biology-related phenotypes. Moreover, these studies vary considerably in terms of their study populations, research designs, measures and other methodological considerations.

#### (i) Role of prenatal conditions

*Animal studies:* Several experimental studies suggest a link between exposure to suboptimal intrauterine conditions such as cortisol, stress or poor diet and shortened offspring telomeres in cells across different tissues [154–158]. For example, a recent study in rodents reported that maternal stress exposure during pregnancy was associated with shorter telomeres in the brain of the adult offspring [158]. In birds, mothers with infection produced offspring with shorter post-hatching TL than non-infected mothers, and there was no effect of paternal infection status, together suggesting a maternally mediated environmental effect [124].

*Human studies:* Several studies in humans have described the effects of prenatal exposures and maternal states and conditions such as obstetric complications, obesity, over- or undernutrition, stress and low socio-economic status during pregnancy, and adverse birth outcomes on offspring telomere biology [159–174]. We have previously reviewed the literature on the role of maternal stress [4,7] and maternal obstetric complications and nutrition during pregnancy [6] in programming offspring telomere biology. Consistent with our framework, many of these obstetric conditions that are related to various aspects of placental or newborn/offspring telomere biology produce perturbations in stress-related oxidative, endocrine, metabolic and immune biological mediators [9]. The majority of these studies have assessed TL or telomerase activity in placenta or cord blood, and only a few studies have examined effects on child or adult telomere dynamics. One of the first studies that examined the long-term effects of adverse intrauterine exposures on later-life telomere length found an association between low birth weight and shorter telomere length in peripheral blood mononuclear cells in preschool aged children [173]. In a study that followed individuals from birth through adulthood, exposure to maternal or perinatal complications was linked to shorter LTL at 38 years of age [138]. In survivors of the siege of Leningrad, exposure to famine during the intrauterine period or childhood was associated with shorter telomere length 70 years after the siege [175]. With reference to prenatal stress, we published the first human study on the long-term effects of maternal psychosocial stress exposure during pregnancy on offspring TL and reported a significant association with LTL in young adult offspring [165]. We and others have since replicated this association between maternal stress during pregnancy and shorter offspring telomere length in several independent cohorts [167,176–178].

### (c) Biological pathways and mechanisms underlying fetal programming of telomere biology

There are no direct neural or vascular connections between the maternal and fetal compartments, and our model proposes that the proximate pathway by which maternal states and conditions during gestation impact embryonic and fetal development is ultimately biological in nature. These biological pathways collectively constitute a process that begins before and around conception and extends through gestation into the postnatal period of life. We propose that the same biological processes that mediate the effects of a range of suboptimal conditions on telomere biology during adult life (reviewed in §3e(i)) also impact fetal programming of the telomere system. Thus, our model focuses particularly on the role of stress-related MPF gestational biology as the key pathways by which maternal states and conditions during pregnancy may programme the offspring telomere biology system. Moreover, we postulate that the mechanisms underlying such programming may be mediated, in part, by the production of stable epigenetic alterations in embryonic and fetal tissues [11]. We also discuss two additional avenues that may be implicated here: trans-generational epigenetic transmission via the germ line, and oocyte biology.

#### (i) Maternal–placental–fetal gestational biology

A substantial body of literature in humans and animals has implicated various maternal–placental–fetal oxidative, immune/inflammatory, endocrine and metabolic pathways in the process of fetal programming of various outcomes [4,8]. However, only a relatively small number of studies to date have examined these pathways in the context of telomere biology-related phenotypes.

*Animal studies*: Animal models have been particularly useful in elucidating the tissue-specific consequences of various experimental manipulations during pregnancy on the offspring's telomere biology system. For example, manipulation of cortisol concentration in the egg yolk of chicken resulted in a higher proportion of short telomeres (and increased levels of reactive oxygen metabolites and prolongation of acute stress response) in the offspring compared to a non-treated control group [154]. In a rodent model, protein restriction *in utero* combined with rapid postnatal catch-up growth (recuperated phenotype) was associated with increased oxidative stress, decreased antioxidant defence mechanisms and accelerated telomere shortening across different tissues in the offspring [155–157,179], and some of these effects persisted in tissues of the reproductive tracts of even second-generation offspring [180]. Post-weaning supplementation with coenzyme Q10, a key component of the electron transport chain and a potent antioxidant, attenuated telomere shortening in leucocytes and aortic cells of recuperated animals [181,182]. Another rodent study of programmed cardiovascular dysfunction indicated that aged offspring of hypoxic pregnancies with maternal antioxidant treatment displayed fewer numbers of short telomeres in vascular tissue compared to offspring of untreated hypoxic pregnancies [183], indicating that therapeutic interventions can be effective in counteracting the detrimental effects of suboptimal intrauterine conditions on cellular ageing.

*Human studies*: Maternal diabetes during pregnancy is an obstetric complication of increasing prevalence and concern. Pregnancies complicated by diabetes exhibit higher oxidative stress in maternal and cord plasma and placental tissue [184]. Gestational diabetes has been associated in a higher percentage of trophoblasts with shortened telomeres [185], shorter newborn LTL [186] and an upregulation of mitochondrial telomerase (TERT) in newborn leucocytes [187]. Another study found that although cord blood TL was not different between offspring from mothers with pre-gestational or gestational diabetes and controls, maternal and newborn glucose concentrations were associated with newborn LTL [188]. Maternal hypertension and its more serious form, pre-eclampsia, represent obstetric complications that confer serious health risks for mother and baby and are related to higher levels of oxidative stress in the mother and child [189]. Hypertensive disorders of pregnancy have been linked to increased expression of placental telomerase mRNA [190] and signs of telomere dysfunction in the placenta and cord blood cells [174,191]. A more recent study reported an association between cord blood levels of dehydroepiandrosterone sulfate (DHEAS), reactive oxygen species and newborn LTL [192]. Finally, yet other studies have reported associations between maternal oestrogen levels [193], folate [166] and vitamin D status during pregnancy [194] with newborn LTL.

#### (ii) Epigenetic alterations in embryonic/fetal tissues

We and others have highlighted the role of epigenetic modifications in the context of intergenerational transmission of maternal effects and fetal programming [11,13,195]. The telomere biology system is under tight epigenetic regulation. Chromatin modifications are key regulators of mammalian telomeres. Sub-telomeric regions are enriched in epigenetic marks that are characteristic of heterochromatin, and the abrogation of master epigenetic regulators such as histone methyltransferases and DNA methyltransferases correlate with loss of TL control (reviewed in [196]). Specifically, the regulation of TL is dependent on the level of methylation in sub-telomeric regions of the histones H3 and H4. The methylation of these histones decreases access to telomere sequences and thus reduces telomerase activity [196]. Hence, proteins such as DNA methyltransferase (that play a role in regulation of methylation) have an impact on TL. Also, DNA methyltransferase is a key candidate mechanism by which early-life conditions such as prenatal nutrition [197] and stress [198] may produce stable, long-term epigenetic alternations. In addition, several studies have suggested that epigenetic modulation of the core promoter region of the TERT gene that regulates telomerase is involved in regulation the telomere maintenance (see [199]). Thus, determining whether these epigenetic mechanisms can potentially be modified by stress-related states and conditions in early life is a future research priority.

#### (iii) Trans-generational epigenetic transmission

Epigenetic alterations in the maternal germ line may provide an avenue for the intergenerational transmission of maternal effects via two possible routes: (*a*) inheritance of maternally derived epigenetic alterations and (*b*) *de novo* production of epigenetic marks in the offspring via exposure to intrauterine conditions [195,200]. With respect to *true* trans-generational epigenetic inheritance, there is currently limited evidence (and only in some animal models) to suggest that epigenetic marks can survive the erasure and re-establishment of epigenetic characteristics that occurs shortly after fertilization [201–204]. Animal models of early-life stress have demonstrated that epigenetic inheritance may be possible through the paternal germ line [205,206], but, to the best of our knowledge, there are yet no studies that have demonstrated such effects through the maternal germ line. However, as discussed in the previous section, it remains highly plausible that *de novo* production of epigenetic alterations in the developing embryo/fetus, via the sequelae of maternal states and conditions, may contribute to the process of fetal/developmental programming

#### (iv) Oocyte cytoplasm and mitochondrial function

The constituents of the oocyte cytoplasm represent the first environmental exposure for a fertilized egg, and variation in oocyte quality significantly affects early embryonic survival, establishment and maintenance of pregnancy, fetal development and even adult disease risk [207–209]. The structure and function of mitochondria, cellular proteins and RNA molecules (e.g. miRNAs) contained in the oocyte cytoplasm are central to these processes [210]. Each of these may, in turn, be impacted by preconception states and conditions at the time of oocyte growth and maturation. For example, maternal obesity prior to conception is associated with altered oocyte endoplasmic reticulum stress signalling [211] and consequently reduced mitochondrial membrane potential and increased autophagy [212]. Empirical evidence from studies of women undergoing *in vitro* fertilization also indicates a significant effect of psychosocial stress [213,214] and physiological stress vulnerability [215] on reduced oocyte competence and failure to conceive. Although alterations in oocyte cytoplasm have not yet been studied in relation to the development of the telomere biology system, it is plausible that the adverse lifelong sequelae of maternal adversity could affect oocyte quality and mitochondrial function across all stages of oocyte development and maturation, contributing to the process of fetal programming of the telomere biology system.

A model integrating telomere biology and mitochondrial function has been suggested by several studies. In fact, a telomere p53–mitochondrion axis may account for many processes that have been implicated in pathophysiological ageing [22]. According to this model, telomere shortening is the driving force that generates mitochondrial dysfunction via activation of the transcription factor p53. Then, mitochondrial dysfunction leads to impaired metabolic as well as energetic homeostasis and increased oxidative stress, which sustains a feed-forward cycle of further DNA damage and mitochondrial dysfunction.

As discussed in a recent review paper [216], the effects of parental stress exposure on offspring telomere length could be directly mediated by parental germ-line telomere length prior to fertilization and its subsequent consequence on the telomere length inherited by the offspring. By contrast, and as described above, indirect effects of parental stress exposure may induce telomere shortening in offspring tissues through increases in maternally derived biological stress mediators during intrauterine life, or through alterations in parental behaviour or care, which then affects offspring stress regulation and thereby induces changes in telomere biology [216].

### (d) Role of postnatal conditions

The influence of postnatal conditions on the characteristics of the telomere biology system has been described in a growing number of studies. For example, several human studies have found that exposure to adverse experiences in infancy and childhood such as abuse and maltreatment, exposure to violence, family disruption and institutionalized care is associated with child TL or TL attrition rate [106,217–225] and leukocyte resting telomerase activity [224]; but see [226–228] for recent reviews and a meta-analysis [229] on this topic. In animals, induction of stress during the early postnatal period (handling and cortisol exposure [230] as well as manipulation of nutrition and begging effort [231] in avian models and maternal separation in a rhesus monkey model [232]) has been shown to produce higher age-related decline of TL during early life [230] and shorter TL in adult life [232].

Our model recognizes that the effects of prenatal and postnatal states and conditions may not be mutually exclusive, and that in many instances the effects of postnatal exposures may, in part, be conditioned upon the effects of prenatal exposures. For example, a recent study reported that early exclusive breastfeeding is associated with longer child telomere length [233]. Prenatal stress exposure is a determinant of breastfeeding behaviour/success [234] as well as of newborn telomere dynamics (as discussed above in §5b(i)). Thus, the likelihood of exposure to certain postnatal conditions, such as reduced breastfeeding, as well as its consequences, such as shorter child telomere length, may be particularly pronounced among individuals exposed to prenatal conditions such as excess stress. It also is possible that the effects of prenatal conditions may be attenuated by other kinds of postnatal experiences such as high maternal and paternal sensitivity and secure attachment patterns.

## 6. Future research directions and conclusion

Based on the conceptual framework and empirical findings presented here, we suggest it is important to consider the potential role of developmental conditions during intrauterine and early postnatal life to arrive at a better understanding of the determinants of the initial setting and function of the telomere biology system and, beyond this, the cellular processes underlying ageing and risk of age-related disorders. Questions and knowledge gaps remain regarding (*a*) the magnitude and duration of the long-term effects of developmental conditions on the initial (newborn) setting of telomere length and telomerase expression and activity; (*b)* the clinical significance of these observed effects on health and disease risk over the lifespan; and (*c*) the precise molecular mechanism(s) underlying the fetal/developmental programming effects on telomere homeostasis. Thus, longitudinal studies are warranted that track the effects of early-life conditions on the telomere biology system from prenatal life and birth onwards through childhood until adulthood and beyond, in order to systematically elucidate their implications in terms of susceptibility for common age-related disorders and longevity.

Given the limitations in humans for performing experimental manipulations of the intrauterine and early postnatal environment and for access to many of the target tissues of interest, appropriate animal models and *in vitro* mechanistic studies are warranted, including studies of stem cells and placental and fetal tissue culture systems and organoids to examine the effects of stress-related oxidative, endocrine, immune and biological processes on telomere biology at various stages of cellular replication and differentiation, as well as their downstream consequences on gene regulatory processes (such as epigenetic characteristics) and cellular energetics (such as mitochondrial function). Moreover, with respect to the putative role of female and male germ cells in the initial setting of offspring telomere dynamics, an intriguing question has recently emerged concerning the relative contribution of factors contained within germ cells as they differentiate, versus the effects of the local micro-environment on germ cells (for example, as established in males by Sertoli cells of the seminiferous epithelium, and in females by Granulosa cells in the primordial follicle) [235].

Methodological barriers to progress in the field of fetal programming of telomere biology include issues related to the reliability of various telomere length measurement approaches [236,237]. The comparability of different methods (e.g. quantitative polymerase chain reaction-based methods, southern blot, fluorescence *in situ* hybridization-based techniques) remains to be established for samples collected from cord blood, placentae and young infants. Furthermore, protocols should be established and harmonized across different laboratories for DNA extraction methods and sample storage conditions. This is particularly important in studies with longitudinal follow-up of the same individuals over time, to reduce possible artefacts such as the observed phenomenon of telomere lengthening that has been attributed to measurement error and short follow-up periods [238].

To conclude, the concepts and findings discussed in this perspectives paper add to the growing appreciation and evidence that the foundations of common, age-related disorders that confer the major societal burden of disease may originate very early in life, and secondly, point to potentially modifiable factors as intervention targets with important implications for primary prevention. The process of fetal programming of the telomere system may represent an important avenue by which population health disparities are propagated across generations to influence the health and well-being of individuals and their offspring across the entire lifespan.

## Data accessibility

This article has no additional data.
